# Novel Hexadeca-Substituted Metal Free and Zinc(II) Phthalocyanines; Design, Synthesis and Photophysicochemical Properties

**DOI:** 10.3390/molecules24010077

**Published:** 2018-12-26

**Authors:** Ayoub I. Awaji, Baybars Köksoy, Mahmut Durmuş, Ateyatallah Aljuhani, Shaya Y. Alraqa

**Affiliations:** 1 Department of Chemistry, Taibah University, Al-Madinah Al Munawrah, P.O. Box 344, Saudi Arabia; a-awaji@hotmail.com (A.I.A.); ateyatallah@hotmail.com (A.A.); 2 Department of Chemistry, Gebze Technical University, 41400 Gebze, Kocaeli, Turkey; baybarsky@gmail.com (B.K.); durmus@gtu.edu.tr (M.D.)

**Keywords:** phthalocyanine, Suzuki-coupling, photophysical, photochemical, photodynamic therapy

## Abstract

The syntheses of a novel 1,4,8,11,15,18,22,25-octahexyloxy-2,3,9,10,16,17,23,24-octa-(4-trifluoromethoxyphenyl) phthalocyanine (**3a**) and its zinc(II) phthalocyanine derivative (**3b**) have been described and characterized by elemental analysis,^1^H NMR, ^13^C NMR, ^19^F NMR, mass, UV-Vis and FT-IR. The newly prepared metal-free phthalocyanine and its zinc(II) counterpart are soluble in most organic solvents. The photophysical and photochemical properties such as aggregation, fluorescence, singlet oxygen generation and photodegradation under light irradiation of these phthalocyanines have been investigated in DMF. The hexadeca-substituted phthalocyanines (**3a** and **3b**) showed longer absorption and emission wavelength values when compared to that of reported phthalocyanine derivatives due to substitution of the all possible positions in the phthalocyanine framework. The zinc(II) phthalocyanine derivative does not only have a good singlet oxygen generation but also has other photophysicochemical properties that enables this phthalocyanine to be useful as a photosensitizer for cancer treatment using photodynamic therapy.

## 1. Introduction

Phthalocyanines (Pcs) are a family of aromatic macrocycles with delocalized 18-π electrons system and are also known as useful functional materials due to their high stability and outstanding chemical and physical properties [1]. These unique properties have lead them to be used in many applications in different scientific and technological areas such as chemical sensors [2], catalysis [3], liquid crystals [4], photodynamic therapy of cancer [5,6], solar energy conversion [7], nonlinear optics [8], semiconductors [9], and optical data storage [10]. Zinc phthalocyanine derivatives are candidates for use as photosensitizing agents in the photodynamic therapy (PDT) of certain cancers because they have long triplet lifetimes and are highly efficient in photogeneration of cytotoxic singlet oxygen [11]. Also, closed-shell and diamagnetic metal ions such as zinc(II), aluminum(III) and silicon(IV) produce metallophthalocyanine derivatives having both long lifetimes and high triplet yields which are also good candidates for photocatalytic applications such as PDT [12]. 

Hexadeca-substituted phthalocyanines are relatively less studied compared to tetra- or octa-substituted counterparts [13,14]. These phthalocyanines are also useful compounds for PDT [15], catalyst [16], optical materials [17], and Langmuir-Blodgett films [18]. Recently these types of phthalocyanines have become a focus of interest for PDT because of their red-shifted absorptions and their existence in one isomeric form (peripheral and non-peripheral tetra phthalocyanines form isomer mixtures) [19,20]. The wavelength of light used in PDT applications is generally within in the range of 550 and 860 nm. The second-generation photosensitizers show high absorption in this wavelength range, and their deep-tissue accumulations are quite high compared to the first generation photosensitizers. Pcs, are known as second generation PDT sensitizers, have excellent photochemical properties due to their high singlet oxygen production capacity and intense absorption in the far red-near IR spectral region (600–850 nm) with a high extinction coefficient. Also, the used light at this wavelength region is not harmful to healthy cells in the body due to their lower energy. In our previously study, the 1,4,8,11,15,18,22,25-octahexyloxy-2,3,9,10,16,17,23,24-octa-(3,5-dichlorophenyl)phthalocyaninato zinc(II) synthesized. The photophysical properties of this zinc(II) phthalocyanine were determined by electronic absorption in the UV-Vis region, fluorescence emission, nanosecond transient absorption and cyclic voltammetry but the PDT properties of this zinc(II) phthalocyanine did not determined [21]. For these reasons, novel hexadeca-substituted metal-free and zinc(II) phthalocyanines bearing hexyloxy groups on the non-peripheral positions and (4-trifluoromethoxyphenyl) groups on the peripheral positions of phthalocyanine ring were designed and synthesized in the present study. The photochemical and photophysical properties of these novel phthalocyanines were also investigated.

## 2. Results and Discussion

### 2.1. Synthesis and Characterization

**Scheme 1:**
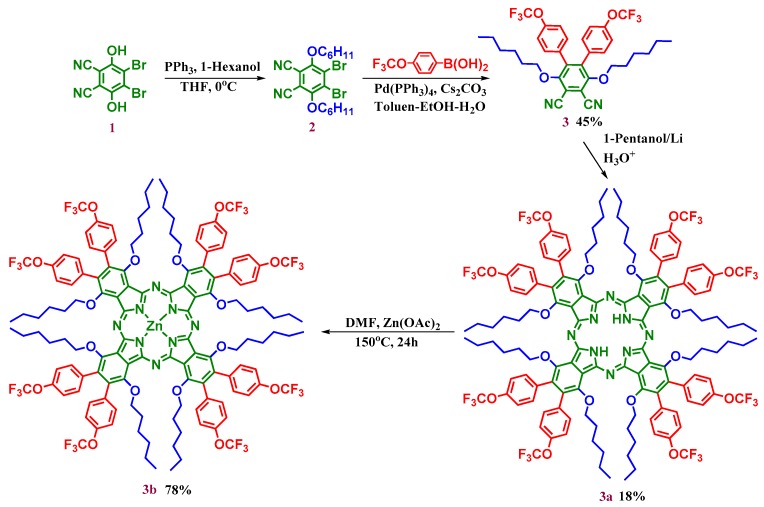
Synthetic pathway of the novel hexadeca-substituted metal-free (**3a**) and zinc(II) (**3b**) phthalocyanines.

The starting compound, 4,5-dibromo-3.6-dihydroxy phthalonitrile, (**1**) was synthesized according to published procedure [22]. Phthalonitrile (**3**) was prepared using the Suzuki coupling reaction where 4,5-dibromo-3,6-dihexyloxyphthalonitrile (**2**) was reacted with 4-trifluoromethoxyphenylboronic acid (Scheme 1). This compound (**3**) was purified by column chromatography resulting in a 45% yield. The target metal-free phthalocyanine (**3a**) was synthesized by the cyclotetramerization of the phthalonitrile (**3**). The zinc(II) phthalocyanine derivative (**3b**) was synthesized by the addition of zinc(II) acetate to the metal-free phthalocyanine derivative (**3a**) (Scheme 1).

These novel phthalocyanines are soluble in common organic solvents such as toluene, dichloromethane, CHCl_3_, THF, and DMF due to the hexadecasubstitution of the phthalocyanine framework with hexyloxy groups on the non-peripheral positions and (4-trifluoromethoxyphenyl) groups on the peripheral positions.

The FT-IR spectra clearly indicated the formation of phthalonitrile (**3**) due to the appearance of C≡N stretching frequency at 2300 cm^−1^. The ^1^H-NMR spectrum showed that all the substituents and ring protons were observed in their expected regions (Figure S1). For phthalonitrile (**3**), the aromatic region displayed two doublet peaks at 7.08 and 7.02 ppm attributed to the four aromatic protons on the phenyl substituents. The ^13^C-NMR spectrum of phthalonitrile (**3**) confirmed that all aromatic and aliphatic carbons are present in their expected regions (Figure S2). The ^19^F-NMR spectrum showed a single peak at −57.83 ppm due to the -OCF_3_ group (Figure S3). The structure of the phthalonitrile (**3**) was also confirmed by MALDI-TOF with the m/z value located at 649.70 as [M + H]^+^ (Figure S4).

**Figure 1:**
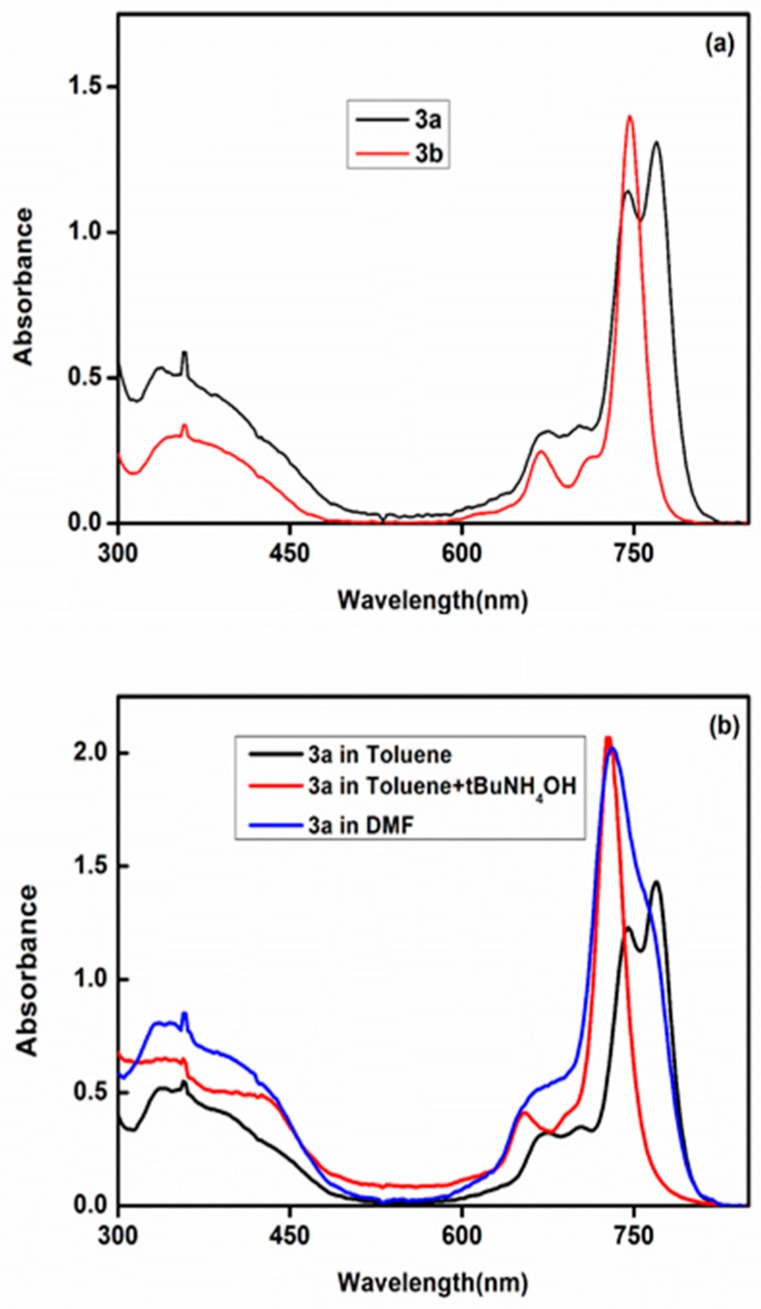
UV-vis absorption spectra of (**a**) **3a** and **3b** in toluene (**b**) **3a** in DMF, toluene and by the addition of *tert*-butylammoniumhydroxide as a base in the toluene solution.

The vibration peak for C≡N stretching of phthalonitrile (**3**) was not show in the FT-IR spectrum of phthalocyanine (**3a**), which provided important support for the formation of the phthalocyanine derivative. The structure of the phthalocyanine (**3a**) was also confirmed by examination of the UV-Vis spectrum because two absorption bands were observed in the Q band region of this phthalocyanine in toluene (Figure 1a). These two Q bands were converted to a single narrow band after addition of zinc(II) metal which is an indicator for the formation of zinc(II) phthalocyanine (**3b**) (Figure 1a). The ^1^H-NMR, ^19^F-NMR and ^13^C-NMR spectra of the novel phthalocyanines (**3a**) and (**3b**) also confirmed the target structures of these phthalocyanines. For ^1^H-NMR spectra, all aromatic and aliphatic protons were observed at their expected regions and integration of the peaks were found to be consistent with the expected numbers of the protons (Figures S5 and S6). The ^13^C-DEPT NMR spectrum of (**3a**) and the ^13^C-NMR spectrum of (**3b**) confirmed that all aliphatic and aromatic carbon atoms appeared at their expected regions (Figures S7 and S8). The ^19^F-NMR spectra showed a single peak at −57.94 ppm for both phthalocyanines (**3a** and **3b**) (Figures S9 and S10). The molecular weight values of the novel phthalocyanines (**3a** and **3b**) were confirmed by MALDI-TOF and m/z values were located at 2596.54 and 2658.54 as [M]^+^ for these phthalocyanines, respectively (Figures S11 and S12).

### 2.2. Electronic Absorption Spectra

Normally, while the metallophthalocyanine derivatives show single narrow Q band absorption in the UV–vis absorption spectra due to their *D_4h_* symmetries, the metal-free phthalocyanines contain two characteristic Q-bands owing to changing symmetries from *D_4h_* to *D_2h_*. But, the UV-Vis absorption spectrum of the studied hexadeca-substituted metal-free phthalocyanine (**3a**) showed a single broad Q band in DMF (Figure 1b) due to consist of a mixture of deprotonated/free base species in this solvent. The deprotonation of the inner hydrogen atoms could be due to formation of hydrogen bond between inner NH group and DMF molecules because of the basicity of DMF solvent (SB = 0.613) [23]. Similar spectra were obtained in other solvents such as DMSO (SB = 0.647) and 1-pentanol (SB = 0.860) [23]. Easy deprotonation of phthalocyanine (**3a**)is probably due to distortion of the macrocycle. Also further proof for deprotonation of the inner –NH protons was obtained by the addition of 0.05 mL 40% tetrabutylammoniumhydroxide as a base to the solution of metal-free phthalocyanine (**3a**) in toluene (Figure 1b). Only one Q band was observed after addition of base to the toluene solution of **3a** indicating that deprotonation occurred in the basic media like in DMF. The UV-Vis electronic absorption spectrum of the zinc(II) phthalocyanine complex (**3b**) showed a characteristic single narrow absorption band in the Q band region at 737 nm in DMF as expected. The molar absorption coefficients values of the compounds (**3a** and **3b**) were found to be 0.98 × 10^5^ L·mol^−1^·cm^−1^ and 1.15 × 10^5^ L·mol^−1^·cm^−1^, respectively. These obtained molar absorption coefficients are consistent with those values for phthalocyanine derivatives given in the literature.

### 2.3. Aggregation Studies

Aggregation is usually depicted as a coplanar association of rings progressing from monomer to dimer and higher order complexes. It is dependent on the concentration, nature of the solvent, nature of the substituents, complexed metal ions and temperature [24]. In this study, the aggregation behavior of hexadeca-substituted metal-free (**3a**) and zinc(II) (**3b**) phthalocyanines were investigated at different concentrations in DMF (Figure S13). The Beer-Lambert law was obeyed for all of these compounds at concentrations ranging from 2–12 μM for determination of the most suitable concentration for further photophysical and photochemical properties of the studied phthalocyanines (**3a** and **3b**). Both hexadeca-substituted phthalocyanines did not show any aggregation at these concentrations range in DMF.

### 2.4. Fluorescence Studies

Figure S14 shows the fluorescence emission spectra of the novel phthalocyanines (**3a** and **3b**). The metal-free phthalocyanine derivative (**3a**) showed two peaks at 742 and 779 nm in DMF due to the formation of protonated and deprotonated species in this solvent. The zinc(II) phthalocyanine (**3b**) showed only one emission peak at 750 nm as expected in DMF solution. 

The fluorescence quantum yields (Φ_F_) of the metal-free (**3a**) and zinc(II) (**3b**) phthalocyanines were determined in DMF. The metal-free phthalocyanine (**3a**) consists of a mixture of deprotonated/free base species in DMF. The obtained Φ_F_ value for this phthalocyanine (**3a**) is related with the mixture of these two species in DMF. The Φ_F_ values of these phthalocyanines were found to be typical of those for previously studied metal-free and zinc phthalocyanines [25]. While the Φ_F_ value of zinc(II) phthalocyanine (**3b**) (Φ_F_ = 0.16) was found similar to the standard unsubstituted zinc phthalocyanine (std-ZnPc) (Φ_F_ = 0.17 [26]), the Φ_F_ value of the metal-free derivative (**3a**) (Φ_F_ = 0.09) was found lower than std-ZnPc in DMF. The τ_F_ values of (**3a** and **3b**) were also determined in DMF using the time correlated single photon counting (TCSPC) technique. The related lifetime spectra for the studied phthalocyanines obtained using this technique are shown in Figure S15. The phthalocyanine derivatives (**3a** and **3b**) exhibited typical mono-exponential decay curves in their lifetime spectra. The τ_F_ value was found 3.17 ns for (**3a**) and 2.03 ns for (**3b**) which were lower than the τ_F_ of the unsubstituted zinc(II) phthalocyanine (τ_F_ = 3.64 ns [26]) in DMF.

### 2.5. Singlet Oxygen Generation Measurements

**Figure 2:**
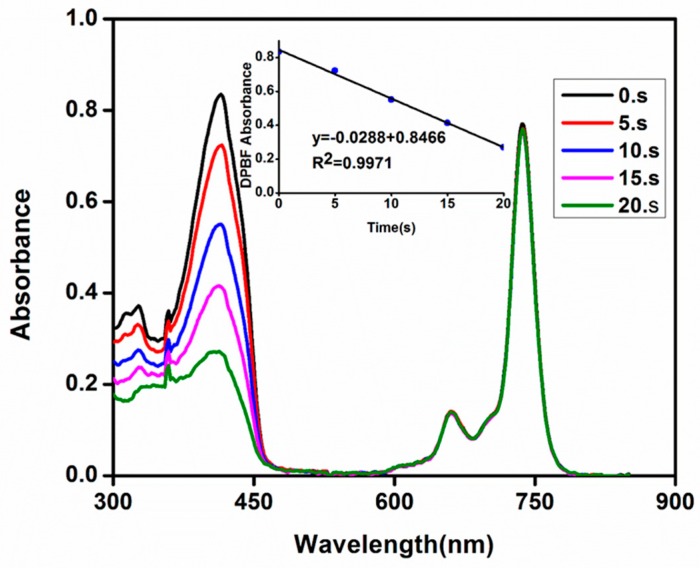
UV-Vis absorption spectral changes for the phthalocyanine **3b** in the presence of DPBF during the determination of singlet oxygen quantum yield at a concentration of 1 × 10^−5^ M in DMSO. Insets: 1,3-Diphenylisobenzofuran (DPBF) absorbances versus time.

Photodynamic therapy (PDT) is a method for treatment of cancer, which uses singlet oxygen generated by a photosensitizer to destroy tumor cells. Pc derivatives are known as second generation photosensitizers for PDT applications due to their high singlet oxygen generation abilities. The photosensitizer molecule is excited to its singlet state and passes to its triplet state through intersystem crossing. The photosensitizer molecule then transfers its energy to the ground state oxygen producing the excited singlet state oxygen which is the chief cytotoxic species for cancer cells. The formed singlet oxygen subsequently kills the tumor cells by a Type II mechanism [27]. The amount of the generated singlet oxygen is quantified as singlet oxygen quantum yield (Φ_Δ_). The Φ_Δ_ values of the novel metal-free (**3a**) and zinc(II) (**3b**) phthalocyanines were determined by using a chemical method for investigation of the photosensitizer ability of phthalocyanines. 1,3-Diphenylisobenzofuran (DPBF) was used as a singlet oxygen trap molecule for the determination of singlet oxygen generated by the novel phthalocyanines (**3a** and **3b**) in DMF. The disappearance of the DPBF absorbance at 417 nm in the presence of the novel phthalocyanines by light irradiation was monitored using a UV–Vis spectrophotometer (Figure 2 for **3b** and S16 for **3a**). The Q band of the samples did not decompose and shifted during light irradiation suggesting that both samples did not degrade by light during singlet oxygen measurements.

The Φ_Δ_ values of the novel metal-free (**3a**) and zinc(II) (**3b**) phthalocyanines were found to be 0.10 and 0.53, respectively in DMF. The obtained Φ_Δ_ value for metal-free phthalocyanine (**3a**) was obtained as the value of the mixture of deprotonated/free base species because this phthalocyanine formed a mixture of these two species in DMF. Both values were lower than standard unsubstituted zinc(II) phthalocyanine (Φ_Δ_ = 0.56 [26]). The zinc(II) phthalocyanine (**3b**) generated more singlet oxygen than its metal-free counterpart (**3a**) due to presence of a closed shell d^10^ zinc(II) metal ion in the cavity of the phthalocyanine (**3b**). It is known that diamagnetic metal ions enhance the singlet oxygen generation ability of the phthalocyanine photosensitizers [27].

### 2.6. Photodegradation Studies

**Figure 3:**
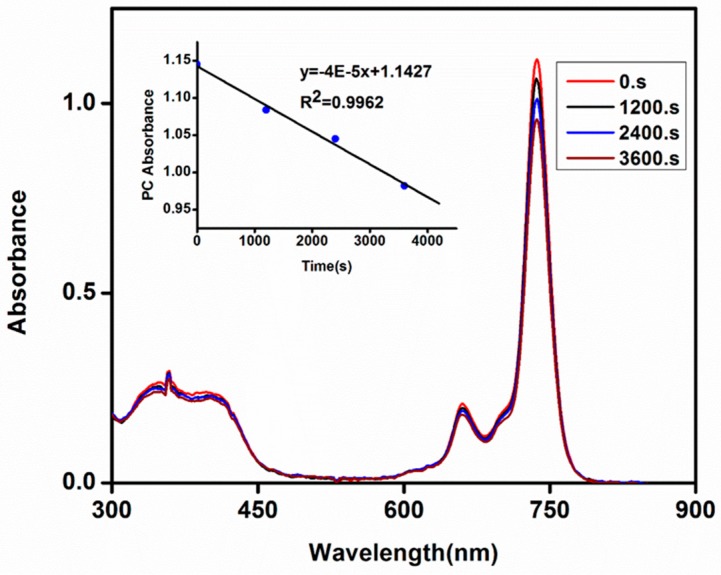
UV-Vis absorption spectral changes during the determination of photodegradation quantum yield for phthalocyanine **3b** in DMSO showing the disappearance of the Q band at 20 minutes intervals. Inset: the plots of the phthalocyanine (Pc) absorbance versus time.

Degradation of the molecules under light irradiation is used to study their stability and this is especially important for those molecules intended for use as photocatalysts. The photodegradation of the studied metal-free (**3a**) and zinc(II) (**3b**) phthalocyanines were determined in DMF. The collapse of the absorption spectra of these phthalocyanines without any distortion of the shape confirms photodegradation is not associated with phototransformation into different forms of phthalocyanines due to light irradiation (Figure 3 for **3b** and S17 for **3a**). The photodegradation rating is determined as photodegradation quantum yields (Φ_d_). The Φ_d_ values are in the order of 10^−4^ for both novel phthalocyanines (**3a** and **3b**) (1.91 × 10^−4^ for **3a** and 2.13 × 10^−4^ for **3b**) and these values are similar to those obtained for phthalocyanine derivatives having different metals in their cavities and substituents on the phthalocyanine ring [11,28]. The Φ_d_ value of metal-free phthalocyanine (**3a**) in DMF was obtained as the value of the mixture of deprotonated/free base species because this phthalocyanine formed a mixture of these two species in this solvent. Stable zinc phthalocyanine molecules show Φ_d_ values as low as 10^−6^ and unstable molecules show these values in the order of 10^−3^ [11]. Both studied phthalocyanine derivatives showed higher Φ_d_ values when compared to unsubstituted zinc(II) phthalocyanine (Φ_d_ = 0.23 × 10^−4^ [26]) suggesting that the substitution of the phthalocyanine macrocycle with hexyloxy groups on the peripheral positions and 4-trifluoromethoxyphenyl groups on the non-peripheral positions of the phthalocyanine framework decreased the stability of the studied phthalocyanine derivatives.

### 2.7. Fluorescence Quenching Studies by 1,4-Benzoquinone

**Figure 4:**
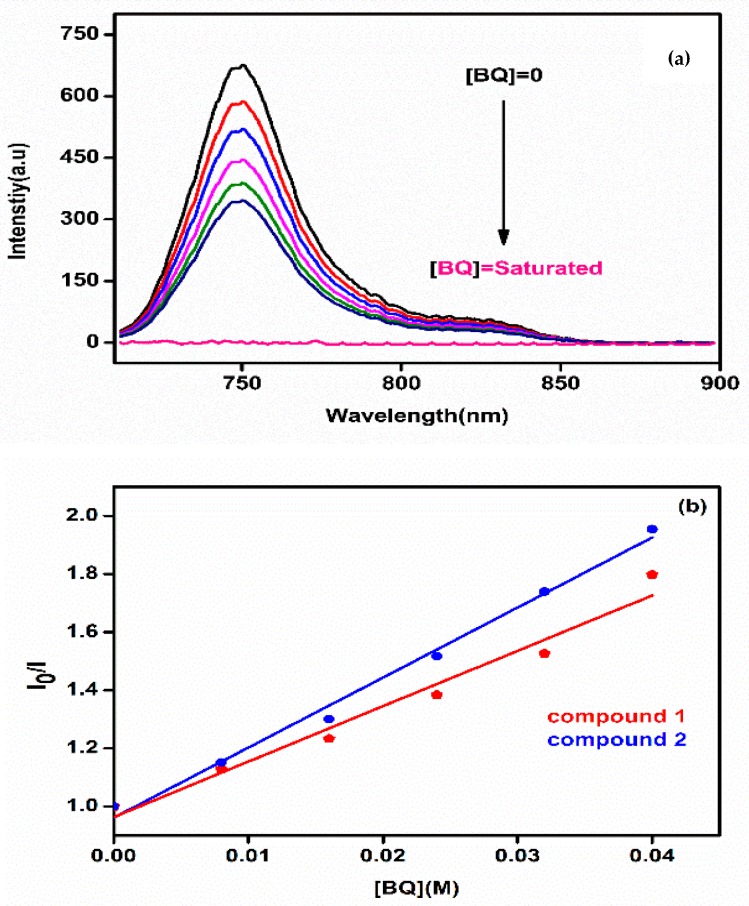
(**a**) Fluorescence emission spectral changes of **3b** (1 × 10^−5^ M) by the addition of different concentrations of BQ in DMF (Excitation wavelength = 700 nm) and (**b**) Stern–Volmer plots for 1,4-benzoquinone (BQ) quenching of introduced metal-free and zinc(II) phthalocyanines. [MPc] = 1 × 10^−5^ M in DMF. [BQ] = 0, 0.008, 0.016, 0.024, 0.032 0.04 M.

The fluorescence quenching behavior of novel metal-free (**3a**) and zinc(II) (**3b**) phthalocyanines by 1,4-benzoquinone (BQ) in DMF was found to obey Stern–Volmer kinetics, which is consistent with diffusion controlled bimolecular reactions. As an example, Figure 4a shows the quenching of (**3b**) by BQ in DMF see Figure S18 for (**3a**)). The slope of the plots shown at Figure 4b gave Stern–Volmer constant (K_SV_) values for the novel hexadeca-substituted phthalocyanines (**3a** and **3b**). These values were found to be 17.82 M^−1^ and 22.79 M^−1^ for phthalocyanines (**3a**) and (**3b**), respectively. The K_SV_ values of the substituted metal-free (**3a**) and zinc(II) (**3b**) phthalocyanines in DMF were lower than unsubstituted zinc(II) phthalocyanine (K_SV_ = 31.90 M^−1^ [26]) suggesting that the substitution decreased the interaction ability of the novel phthalocyanines to BQ. The bimolecular quenching rate constant (k_q_) values of the substituted phthalocyanines (**3a** and **3b**) were also determined and they found to be 0.56 × 10^10^ M^−1^·s^−1^ for (**3a**) and 1.12 × 10^10^ M^−1^·s^−1^ for (**3b**) in DMF. The k_q_ values of the substituted phthalocyanines were also found lower than unsubstituted zinc(II) phthalocyanine (k_q_ = 2.61 × 10^10^ M^−1^·vs^−1^ [26]). The k_q_ values were found to be close to the diffusion-controlled limits, ~ 10^10^ M^−1^·s^−1^, which agrees with the Einstein–Smoluchowski approximation at room temperature for diffusion-controlled bimolecular interactions [29].

## 3. Materials and Methods

### 3.1. Materials

All used chemicals and solvents were of reagent grade quality. The starting compound, 4,5-dibromo-3.6-dihydroxy phthalonitrile (**1**) was synthesized according to published procedure [22]. *p*-Trifluoromethoxyophenyl boronic acid, zinc(II) acetate, Cs_2_CO_3_ and Pd(PPh_3_)_4_ were purchased from Sigma-Aldrich (St. Louis, MO, USA) and they were used as received. 1,3-Diphenylisobenzofuran (DPBF) was purchased from Fluka (St. Louis, MO, USA). The used solvents were purified, dried and stored over molecular sieves (4Å). All reactions were carried out under dry nitrogen atmosphere unless otherwise noted. Column chromatography was performed on silica gel 60 for a proper purification of the crude compound. Melting points of the phthalocyanine compounds were found to be higher than 300 °C. The purity of the products was tested in each step by thin layer chromatography (Silica gel F-254 coated TLC plate). 

### 3.2. Equipment

FT-IR spectra were recorded using KBr sample disks on a Schimadzu Fourier Transform Infrared Spectrometer FTIR-400 (San Jose, CA, USA) and a Perkin Elmer Spectrum 100 spectrometer (Waltham, MA, USA). ^1^H, ^13^C and ^19^F-NMR spectra of phthalocyanine derivatives were recorded on a Bruker AVANCE 400 MHz spectrometer (Madison, MI, USA). UV-Vis spectra were recorded on a Schimadzu 2101 UV/Vis spectrometer (Kyoto, Japan). Mass spectra were acquired on Microflex MALDI-TOF mass spectrometer (Bruker Daltonics MS, Bremen, Germany) equipped with a nitrogen UV-Laser operating at 337 nm and the spectra recorded in reflectron mode with average of 50 shots using 2,5-dihydroxybenzoicacid (DHB) as a MALDI matrix. The samples for matrix-assisted laser desorption/ionization (MALDI) were prepared by mixing sample solutions (1 mg/mL in DMF) and the matrix solution (1:10 *v*/*v*) in a 0.5 mL Eppendorf^®^ micro tube (Hamburg, Germany). Finally, 0.5 μL of this mixture was deposited on the sample plate, dried at room temperature, and then analyzed. Elemental analyses were performed by the Thermo Finnigan Flash 1112 Instrument (Waltham, MA, USA). Fluorescence excitation and emission spectra (non-corrected) were recorded on a Varian Eclipse spectrofluorometer using a 1 cm path length cuvette at room temperature. Photo-irradiations were done using a General Electric quartz line lamp (300W). A 600 nm glass cut off filter (Schott) and a water filter were used to filter off ultraviolet and infrared radiations, respectively. An interference filter (Intor, 700 nm with a band width of 40 nm) was additionally placed in the light path before the sample. Light intensities were measured with a POWER MAX5100 (Los Angeles, CA, USA, Molelectron detector incorporated) power meter.

### 3.3. Photophysical and Photochemical Parameters

#### 3.3.1. Fluorescence Quantum Yields and Lifetimes

Fluorescence quantum yields (Φ_F_) were determined by the comparative method using Equation (1) [30],

(1) $$\Phi F=\Phi F\left(\mathrm{Std}\right)\frac{{F\times A_{\mathrm{Std}}\times n}^{2}}{F{{}_{\mathrm{Std}}\times A\times n}_{\mathrm{Std}}^{2}}$$

where F and F_Std_ are the areas under the fluorescence emission curves of the samples (**3a** and **3b**) and the standard, respectively. A and A_Std_ are the respective absorbances of the samples (**3a** and **3b**) and standard at the excitation wavelengths, respectively. n^2^ and $n_{\mathrm{Std}}^{2}$ are the refractive indices of solvents used for the samples (**3a** and **3b**) and standard, respectively. Unsubstituted ZnPc (Φ_F_ = 0.17 in DMF) [31] was employed as the standard. Fluorescence lifetimes were measured by a time correlated single photon counting (TCSPC) method using FLUOROLOG-3 spectrofluorometer (Horiba JobinYvon, Edison, NJ, USA) equipped with a NanoLED and a standard air cooled R928 PMT detector.

#### 3.3.2. Singlet Oxygen Quantum Yields

Singlet oxygen quantum yields (Φ_Δ_) of phthalocyanine compounds (**3a** and **3b**) were carried out using the experimental set-up described in the literature [26,27]. Typically, a 3 mL portion of phthalocyanine solutions (C = 1 × 10^−5^ M) containing the singlet oxygen quencher were irradiated in the Q band region with the photo-irradiation set-up described in references [26,27]. Singlet oxygen quantum yields (Φ_Δ_) were determined in air using the relative method using unsubstituted ZnPc (in DMF) as a reference. DPBF was used as a chemical quencher for singlet oxygen in DMF. Equation (2) was employed for the calculations:

(2) $$\Phi \Delta =\Phi_{\Delta }^{\mathrm{Std}}\frac{{R\times I}_{\mathrm{abs}}^{\mathrm{Std}}}{R^{\mathrm{Std}}\times I_{\mathrm{abs}}}$$

The $\Phi_{\Delta }^{\mathrm{Std}}$ is the singlet oxygen quantum yield for the standard unsubstituted ZnPc (Φ_Δ_ = 0.56 in DMF) [26]. R and R_Std_ are the DPBF photobleaching rates in the presence of the respective samples (**3a** and **3b**) and standard, respectively. I_abs_ and $I_{\mathrm{abs}}^{\mathrm{Std}}$ are the rates of light absorption by samples (**3a** and **3b**) and the standard, respectively. To avoid chain reactions induced by DPBF in the presence of singlet oxygen, the concentration of the quencher (DPBF) was lowered to ∼3 × 10^−5^ M [32]. Solutions of the sensitizer and the standard (C = 1 × 10^−5^ M) containing DPBF were prepared in the dark and irradiated in the Q band region using the photo-irradiation setup. DPBF degradation at 417 nm was monitored. A light intensity of 6.60 × 10^15^ photons s^−1^ cm^−2^ was used for Φ_Δ_ determinations.

#### 3.3.3. Photodegradation Quantum Yields

Photodegradation quantum yield (Φ_d_) determinations were carried out using the experimental set-up described in literature [26,27]. Photodegradation quantum yields were determined using Equation (3),

(3) $$\Phi_{d}=\frac{(C_{0}-C_{t})\times V\times N_{A}}{I_{\mathrm{abs}}\times S\times t}$$

The C_0_ and C_t_ are the concentrations of phthalocyanine compounds (**3a** and **3b**) before and after irradiation, respectively, V is the reaction volume, N_A_ is Avogadro’s constant, S is the irradiated cell area, t is the irradiation time and I_abs_ is the overlap integral of the radiation source light intensity and the absorption of the samples (**3a** and **3b**). A light intensity of 2.20 × 10^16^ photons s^−1^ cm^−2^ was employed for Φ_d_ determinations. 

#### 3.3.4. Fluorescence Quenching by 1,4-Benzoquinone (BQ)

Fluorescence quenching experiments on the substituted metal-free and zinc(II) phthalocyanines (**3a** and **3b**) were carried out by the addition of different concentrations of BQ to a fixed concentration of the phthalocyanines, and the concentrations of BQ in the resulting mixtures were 0, 0.008, 0.016, 0.024, 0.032 and 0.040 M. The fluorescence spectra of substituted metal-free and zinc(II) phthalocyanines (**3a** and **3b**) at each BQ concentration were recorded, and the changes in fluorescence intensity related to BQ concentration were determined by the Stern–Volmer (SV) Equation [33] (Equation (4)):

(4) $$\frac{I_{O}}{I}=1+K_{\mathrm{SV}}\left[\mathrm{BQ}\right]$$

The I_o_ and I are the fluorescence intensities of fluorophore in the absence and presence of quencher, respectively. K_SV_ is the Stern–Volmer constant; and this is the product of the bimolecular quenching constant (k_q_) and the fluorescence lifetime (τ_F_) (Equation (5)):

K_SV_ = k_q_ × τ_F_ (5)

The ratios I_o_/I were calculated and plotted against [BQ] according to Equation (4), and K_SV_ determined from the slope.

### 3.4. Synthesis

#### 3.4.1. 4,5-Bis(4-trifloromethoxyphenyl)-3,6-bis(hexyloxy)phthalonitrile (**3**)

4,5-Dibromo-3,6-dihexyloxyphthalonitrile (100 mg, 0.205 mmol), 4-(trifluoromethoxy) phenyl boronic acid (127 mg, 0.615 mmol), tetrakis (triphenylphosphine) palladium(0) complex (118 mg, 0.102 mmol) and cesium carbonate (668 mg, 2.1 mmol) as a base were added to a solution of toluene, ethanol and water (*v*/*v*/*v*, 3:3:1) solvent mixture. The reaction mixture was heated at refluxing temperature for 24 h with stirring under a nitrogen atmosphere. Then the solvent mixture was removed under vacuum using rotary evaporator. The target phthalonitrile was isolated by column chromatography with silica gel using hexane:THF (*v*/*v*, 10:1) solvent system as an eluent. Finally, the product was recrystallized from ethanol. Yield: 59.8 mg (45%). M.p. 150 °C. FT-IR [ATR ν_max_/cm^−1^]: 3039 (Aromatic-CH), 2940-2856 (Aliphatic-CH), 2300 (C≡N), 1562 (Aromatic-C=C), 900 (C–F). ^1^H-NMR (400 MHz, CDCl_3_) δ 7.08 (d, *J* = 8.6 Hz, 4H, Aromatic-CH), 7.02 (d, *J* = 8.6 Hz, 4H, Aromatic-CH), 3.69 (t, *J* = 6.4 Hz, 4H, Aliphatic-CH), 1.42-1.33 (m, 4H, Aliphatic-CH), 1.16-1.06 (m, 4H, Aliphatic-CH), 1.05-0.98 (m, 8H, Aliphatic-CH), 0.8 (t, *J* = 7.06 Hz, 6H, Aliphatic-CH). ^13^C-NMR (100 MHz, CDCl_3_) δ: 156.5 (Aromatic-C), 148.9 (Aromatic-C), 141.2 (Aromatic-C), 131.9 (Aromatic-C), 131.8 (Aromatic-C), 120.4 (Aromatic-C), 120.3 (1C, q, *J_C–F_* = 259 Hz, CF_3_)., 113.0 (Aromatic-C), 109.8 (Aromatic-C), 76.03 (Aliphatic-OCH_2_), 31.2 (Aliphatic-CH_2_), 29.6 (Aliphatic-CH_2_), 25.1 (Aliphatic-CH_2_), 22.3 (Aliphatic-CH_2_), 13.8 (Aliphatic-CH_3_). ^19^F- NMR (377 MHz, CDCl_3_) δ -57.83 (C–F), Mass (MALDI-TOF): m/z, Calcd. for C_34_H_34_N_2_F_6_O_4_; 648.64 g/mol, found; 649.70 [M + H]^+^. Anal Calc. for C_34_H_34_N_2_F_6_O_4_: C, 62.96%; H, 5.28%; N, 4.32%. Found: C, 63.08%; H, 5.14%; N, 4.26%.

#### 3.4.2. 1,4,8,11,15,18,22,25-Octakis(hexyloxy)-2,3,9,10,16,17,23,24-octakis(4-trifloro methoxyphenyl) phthalocyanine (**3a**)

4,5-Bis(4-trifloromethoxyphenyl)-3,6-bis(hexyloxy) phthalonitrile (**3**) (100 mg, 0.154 mmol) was dissolved in 1-pentanol and heated at 160 °C for 30 min, and then a small piece of Li was added to the solution under argon atmosphere. Then, the reaction mixture was cooled to the room temperature then a few drops of acetic acid were added to the solution to remove the metal (Li). The methanol was added to precipitate the target phthalocyanine compound. Finally, a further purification was done by silica gel column chromatography using hexane/ethyl acetate (*v*/*v*, 1:1) solvent system as an eluent. Yield: 9 mg (18%). M.p. > 300 °C. FT-IR [ATR ν_max_/cm^−1^]: 3480 (-NH), 3037 (Aromatic-C-H), 2964-2854 (Aliphatic C-H), 1540 (Aromatic-C=C). UV/Vis (DMF) λ_max_/nm (log ε): 339 (4.59), 397 (4.51), 665 (4.40), 731 (4.99). ^1^H-NMR (400 MHz, CDCl_3_) δ 7.34 (d, *J* = 8.1 Hz, 16H, Aromatic-CH), 7.11 (d, *J* = 8.1 Hz, 16H, Aromatic-CH), 4.64 (bs, 16H, Aliphatic-CH), 1.66-1.56 (m, 16H, Aliphatic-CH), 1.18-0.98 (m, 48H, Aliphatic-CH), 0.81-0.74 (m, 24H, Aliphatic-CH), 0.34 (s, 2H, NH). ^13^C-DEPT135 NMR (400 MHz, CDCl_3_) δ: 133.3 (CH aromatic), 120.0 (CH aromatic), 76.8 (OCH_2_), 31.8 (CH_2_), 30.1 (CH_2_), 25.8 (CH_2_), 25.5 (CH_2_), 14.0 (CH_3_), ^19^F- NMR (377 MHz, CDCl_3_) δ -57.94 (C–F), Mass (MALDI-TOF): *m/z*: Calcd. for C_136_H_138_N_8_F_24_O_16_; 2596.57 g/mol, found 2596.54 [M]^+^. Anal Calc. for C_136_H_138_N_8_F_24_O_16_: C, 62.91%; H, 5.36%; N, 4.32%. Found: C, 63.11%; H, 5.45%; N, 4.40%.

#### 3.4.3. 1,4,8,11,15,18,22,25-Octakis(hexyloxy)-2,3,9,10,16,17,23,24-octakis(4-trifloro methoxyphenyl) phthalocyaninato zinc(II) (**3b**)

15 mg (0.00578 mmol) metal-free phthalocyanine derivative **3a** was dissolved in 5 mL dry DMF and 2.12 mg (0.01156 mmol) zinc(II) acetate was added to this solution. This reaction mixture was stirred and heated at 150 °C about 12 h under argon atmosphere and then poured into water after cooling to room temperature and centrifuged. The crude product was purified by column chromatography using dichloromethane as an eluent. Yield: 12 mg (78%). FT-IR [ATR ν_max_/cm^−1^]: 3039 (Aromatic-C-H), 2956-2856 (Aliphatic C-H), 1512 (Aromatic-C=C). UV/Vis (DMF) λ_max_/nm (log ε): 345 (4.45), 398 (4.33), 654 (4.33), 737 (5.06). ^1^H-NMR (400 MHz, CDCl_3_) δ 7.45 (d, *J* = 8.1 Hz, 16H, Aromatic-CH), 7.19 (d, *J* = 8.1 Hz, 16H, Aromatic-CH), 4.80 (bs, 16H, Aliphatic-CH), 1.73-1.67 (m, 16H, Aliphatic-CH), 1.32-1.08 (m, 48H, Aliphatic-CH), 0.91–0.84 (m, 24H, Aliphatic-CH). ^13^C-NMR (100 MHz, CDCl_3_) δ: 152.4 (Aromatic-C), 148.1 (Aromatic-C), 137.2 (Aromatic-C), 133.1 (Aromatic-C), 132.2 (Aromatic-C), 130.8 (Aromatic-C), 119.6 (1C, q, *J_C–F_* = 259 Hz, CF_3_), 110.2 (Aromatic-C), 109.7 (Aromatic-C), 73.7 (Aliphatic-CH_2_), 31.8 (Aliphatic-CH_2_), 29.7 (Aliphatic-CH_2_), 25.8 (Aliphatic-CH_2_), 22.5 (Aliphatic-CH_2_), 13.8 (Aliphatic-CH_2_). ^19^F- NMR (377 MHz, CDCl_3_) δ -57.94 (C–F), Mass (MALDI-TOF): *m/z*: Calcd. for C_136_H_136_N_8_F_24_O_16_Zn; 2658.35 g/mol, found 2658.54 [M]^+^. Anal Calc. for C_136_H_136_N_8_F_24_O_16_Zn: C, 61.41%; H, 5.15%; N, 4.21%. Found: C, 61.34%; H, 5.08%; N, 4.41%. 

## 4. Conclusions

In conclusion, the novel hexadeca-substituted metal-free (**3a**) and zinc(II) (**3b**) phthalocyanines bearing 4-trifluoromethoxyphenyl groups on the peripheral positions and hexyloxy groups on the non-peripheral positions of the phthalocyanine macrocycle were synthesized. While 4-trifluoromethoxyphenyl groups were introduced by the Suzuki-Miyaura coupling reaction, hexyloxy groups were attached to phthalocyanine ring by the Mitsunobu reaction. The novel phthalocyanines were characterized by elemental analysis, ^1^H NMR, ^13^C NMR, ^19^F NMR, mass, UV-Vis and FT-IR. The photophysical and photochemical properties such as aggregation, fluorescence quantum yields and lifetimes, singlet oxygen generation and photodegradation under light irradiation of these novel phthalocyanines were also investigated in DMF. These properties were determined as a mixture of deprotonated/free base species of metal-free phthalocyanine (**3a**) in DMF because this phthalocyanine formed a mixture of these two species in DMF. Both studied phthalocyanines showed excellent solubility in common organic solvents such as toluene, dichloromethane, chloroform, THF and DMF. These phthalocyanines exhibited light absorption at long wavelength in the UV-Vis spectra due to the hexadeca-substitution. As a result of the photophysical and photochemical properties, the metal free phthalocyanine (**3a**) and especially the zinc(II) phthalocyanine derivative (**3b**) can be considered as candidates for photosensitizers in the treatment of cancer using the PDT technique due to the high singlet oxygen generation ability of the phthalocyanine derivatives. In vitro cytotoxicity properties and photodynamic activities of the studied phthalocyanines, especially(**3b**), will be tested against different cancer cell lines including different carcinoma cells lines in future work.

## Supplementary Materials

The following are available online. Figure S1: ^1^H-NMR spectrum of compound **3** in CDCl_3_, Figure S2: ^13^C-NMR spectrum of compound **3** in CDCl_3_, Figure S3: ^19^F-NMR spectrum of compound **3** in CDCl_3_, Figure S4: MALDI-TOF spectrum of compound **3** in CDCl_3_, Figure S5: ^1^H-NMR spectrum of compound **3a** in CDCl_3_, Figure S6: ^1^H-NMR spectrum of compound **3b** in CDCl_3_, Figure S7: ^13^C-NMR spectrum of compound **3a** in CDCl_3_, Figure S8: ^13^C-NMR spectrum of compound **3b** in CDCl_3_, Figure S9: ^19^F-NMR spectrum of compound **3a** in CDCl_3_, Figure S10: ^19^F-NMR spectrum of compound **3b** in CDCl_3_, Figure S11: MALDI-TOF spectrum of compound **3a** in CDCl_3_, Figure S12: MALDI-TOF spectrum of compound **3b** in CDCl_3_, Figure S13: UV–vis spectra of (a) **3a** and (b) **3b** in DMF at different concentration (C=2–12µM), Figure S14: Fluorescence emission spectra of (a) phthalocyanine **3a** and (b) phthalocyanine **3b** in DMF at 5 × 10^−6^ M. (Excitation wavelength = 686 nm for **3a** and 700 nm for **3b**), Figure S15: Time correlated single photon counting (TCSPC) trace for (a) **3a** (Excitation wavelength = 686 nm) and (b) **3b** (Excitation wavelength = 700 nm) in DMF with residuals, Figure S16: The electronic absorption spectral changes during the determination of singlet oxygen quantum yields. This determination was for **3a** in DMF at a concentration of 1 × 10^−5^ M. (Inset: Plot of DPBF absorbances versus time), Figure S17: The electronic absorption spectral changes of **3a** in DMF under light irradiation showing the disappearance of the Q-band (Inset: plot of phthalocyanine absorbances versus time), Figure S18: Fluorescence emission spectral changes of **3a** (1 × 10^–5^ M) by the addition of different concentrations of BQ in DMF.
