# Supplementary material for: Novel Hexadeca-Substituted Metal Free and Zinc(II) Phthalocyanines; Design, Synthesis and Photophysicochemical Properties
Source: Molecules. 2018 Dec 26;24(1):77. doi: 10.3390/molecules24010077 (PMC6337579; doi:10.3390/molecules24010077)
Supplement: Supplementary file 1 [file molecules-24-00077-s001.pdf]

Supporting Information

Novel hexadeca-substituted metal free and zinc(II) phthalocyanines; Design, synthesis and photophysicochemical properties

Ayoub Ibrahim Awaji[a], Baybars Köksoy[b], Mahmut Durmuş[b]*, Ateyatallah Al-Juhani[a], Shaya Y. Al-Raqa[a]*

[a]*Taibah University, Departmen of Chemistry, P.O Box 344,* Al-Madinah Al Munawrah, Saudi Arabia

[b]*Gebze Technical University, Department of Chemistry,* Gebze 41400, Kocaeli, Turkey

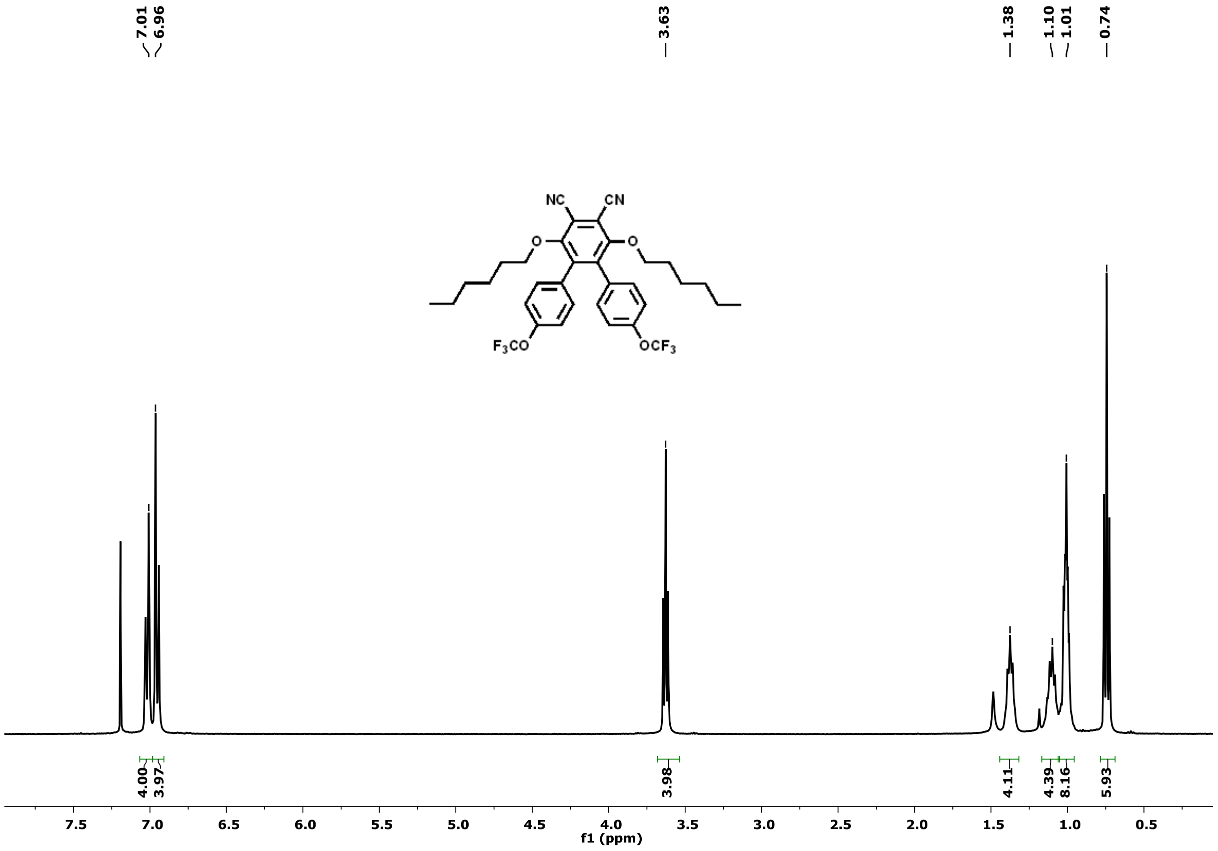

Figure S1. ^{1}H-NMR spectrum of compound **3** in $CDCl_3$.

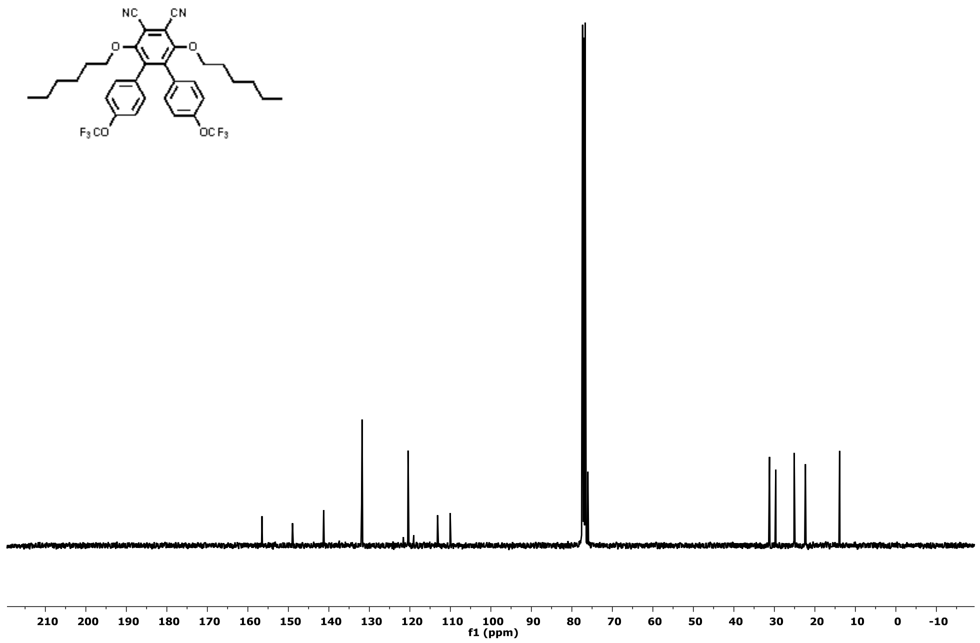

Figure S2. ^{13}C-NMR spectrum of compound **3** in $CDCl_3$.

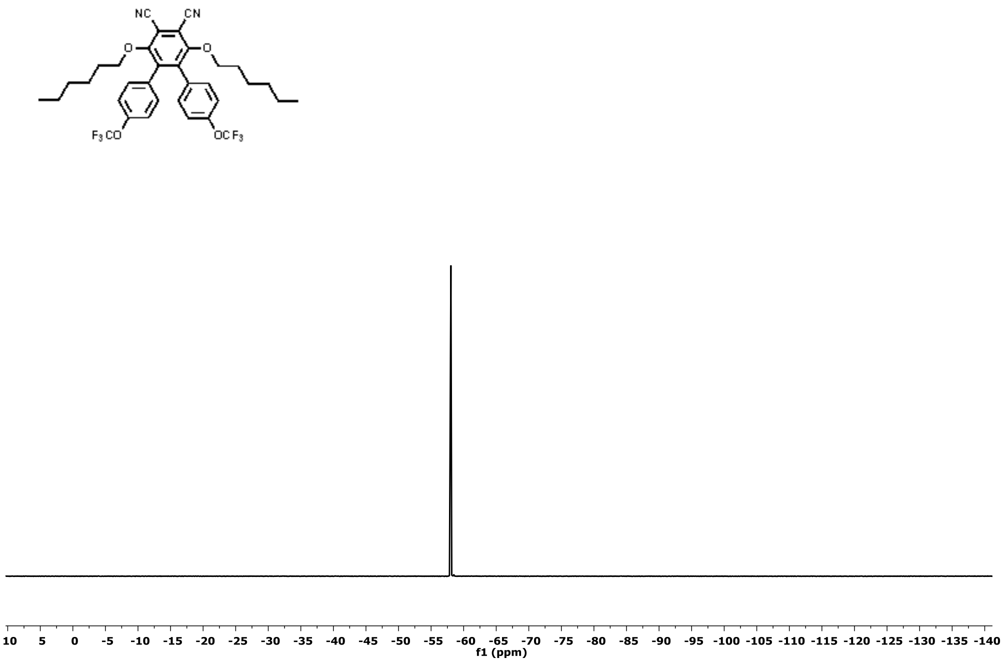

Figure S3. ^{19}F-NMR spectrum of compound **3** in $CDCl_3$.

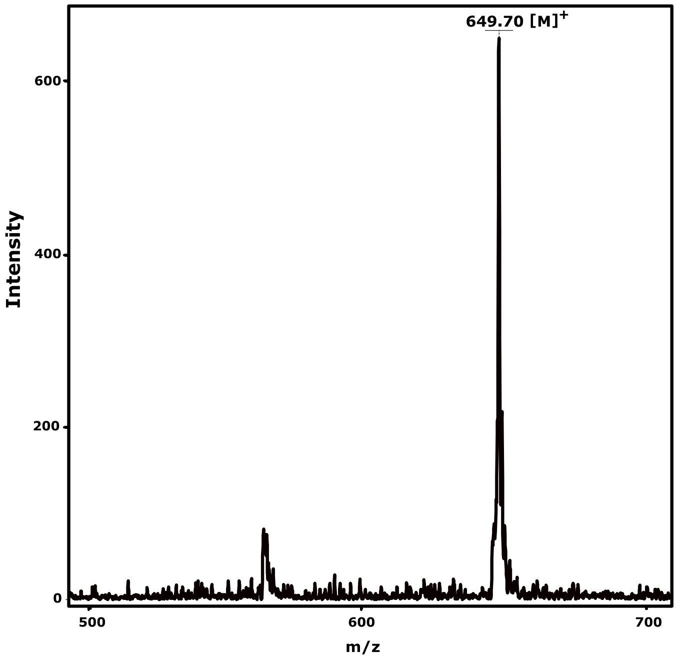

Figure S4. MALDI-TOF spectrum of compound **3** in $CDCl_3$.

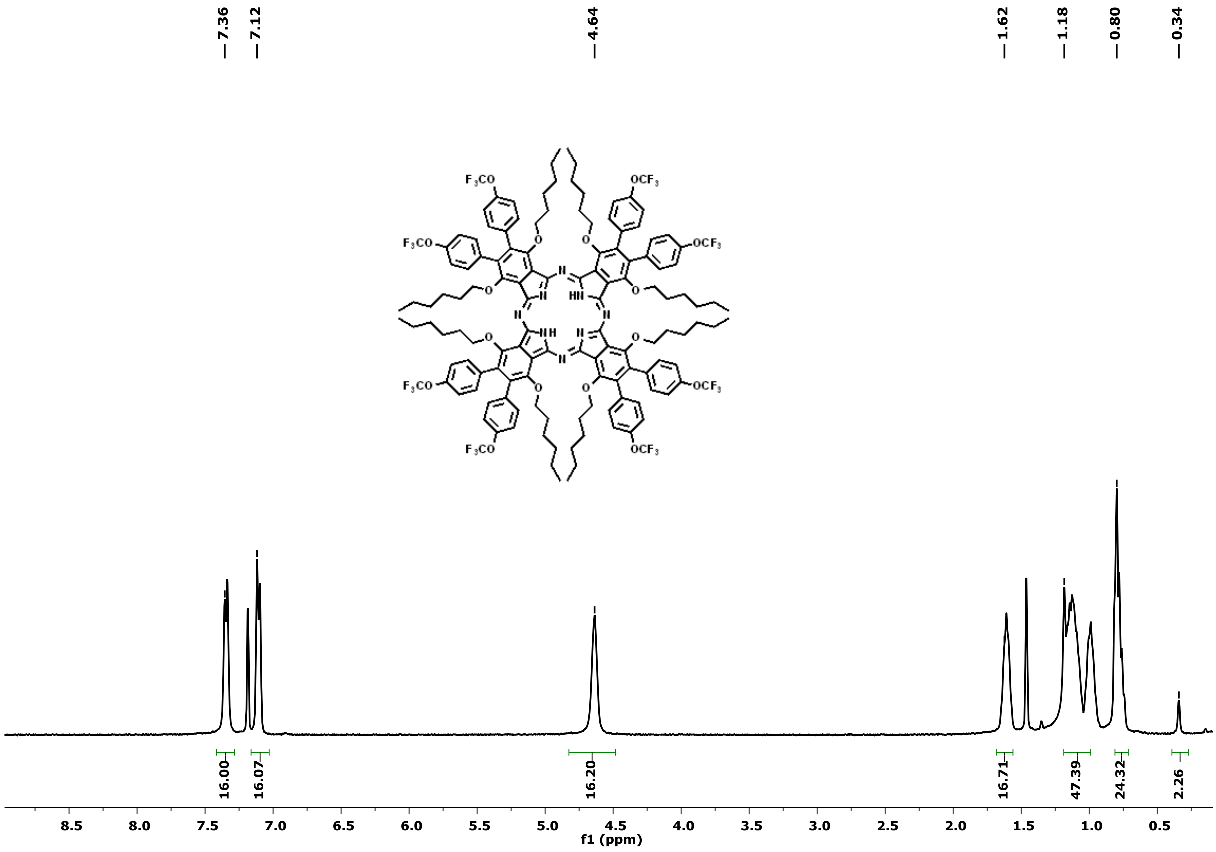

Figure S5. ^{1}H-NMR spectrum of compound **3a** in $CDCl_3$.

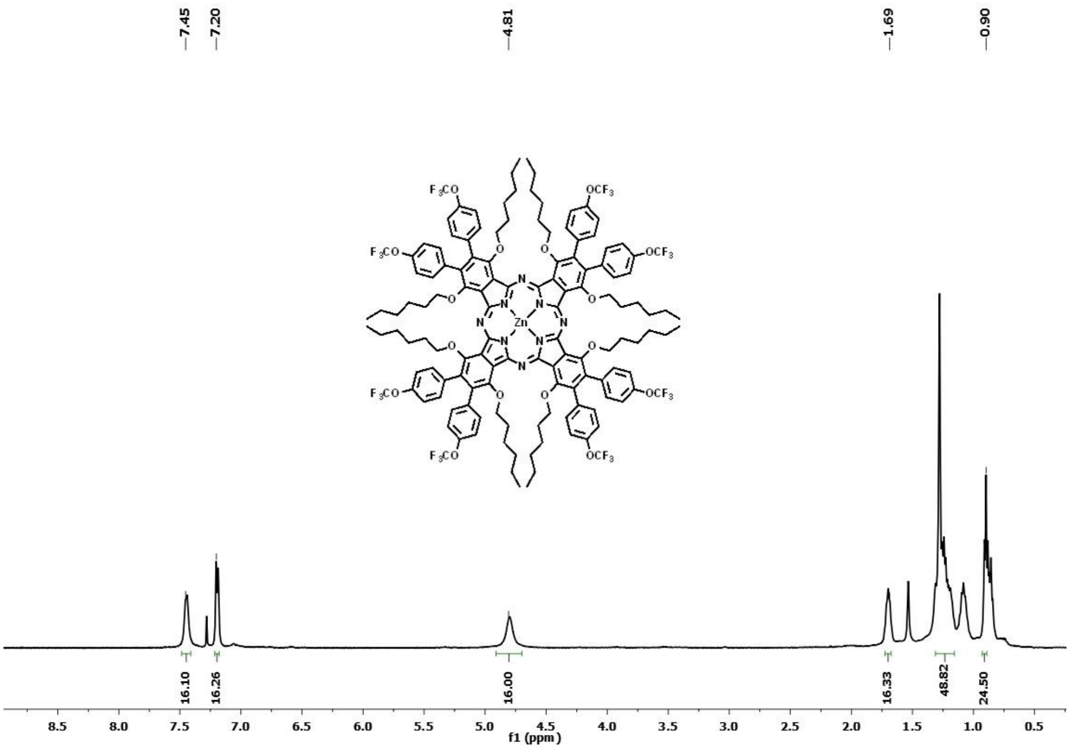

Figure S6. ^{1}H-NMR spectrum of compound **3b** in $CDCl_3$.

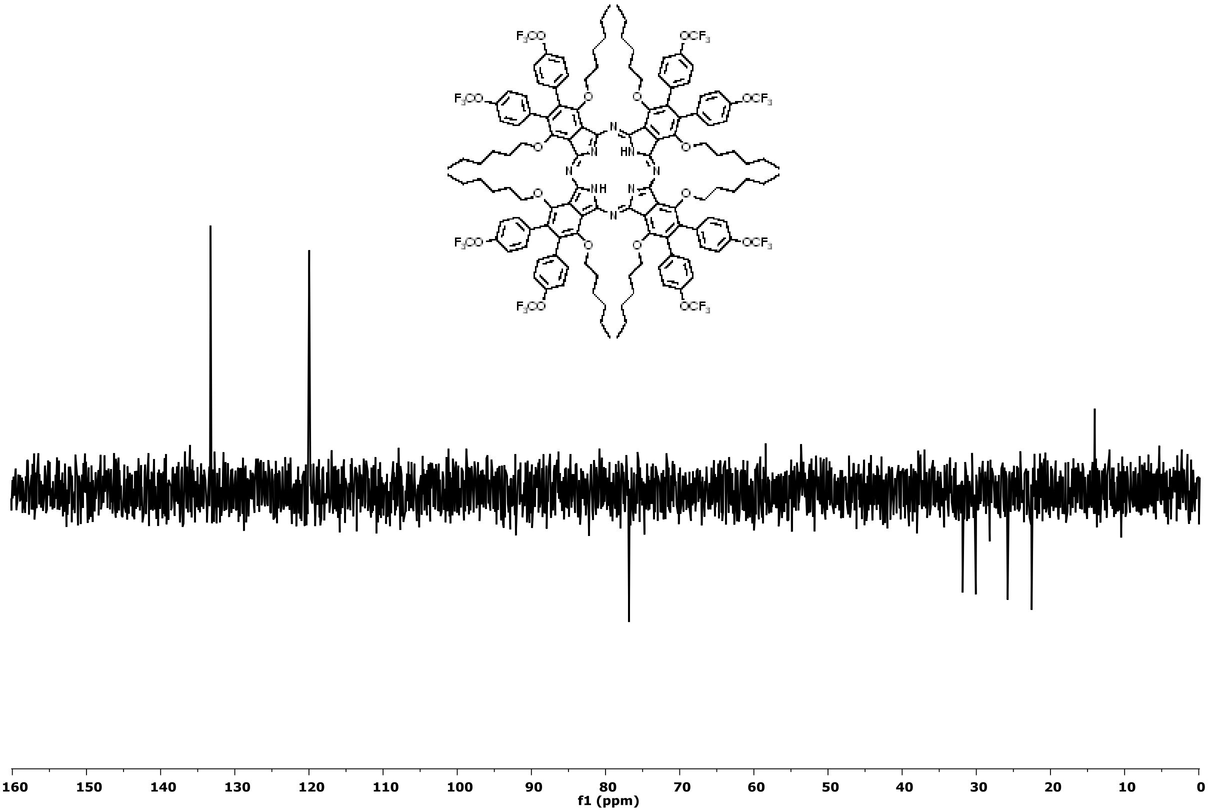

Figure S7. ^{13}C-NMR spectrum of compound **3a** in $CDCl_3$.

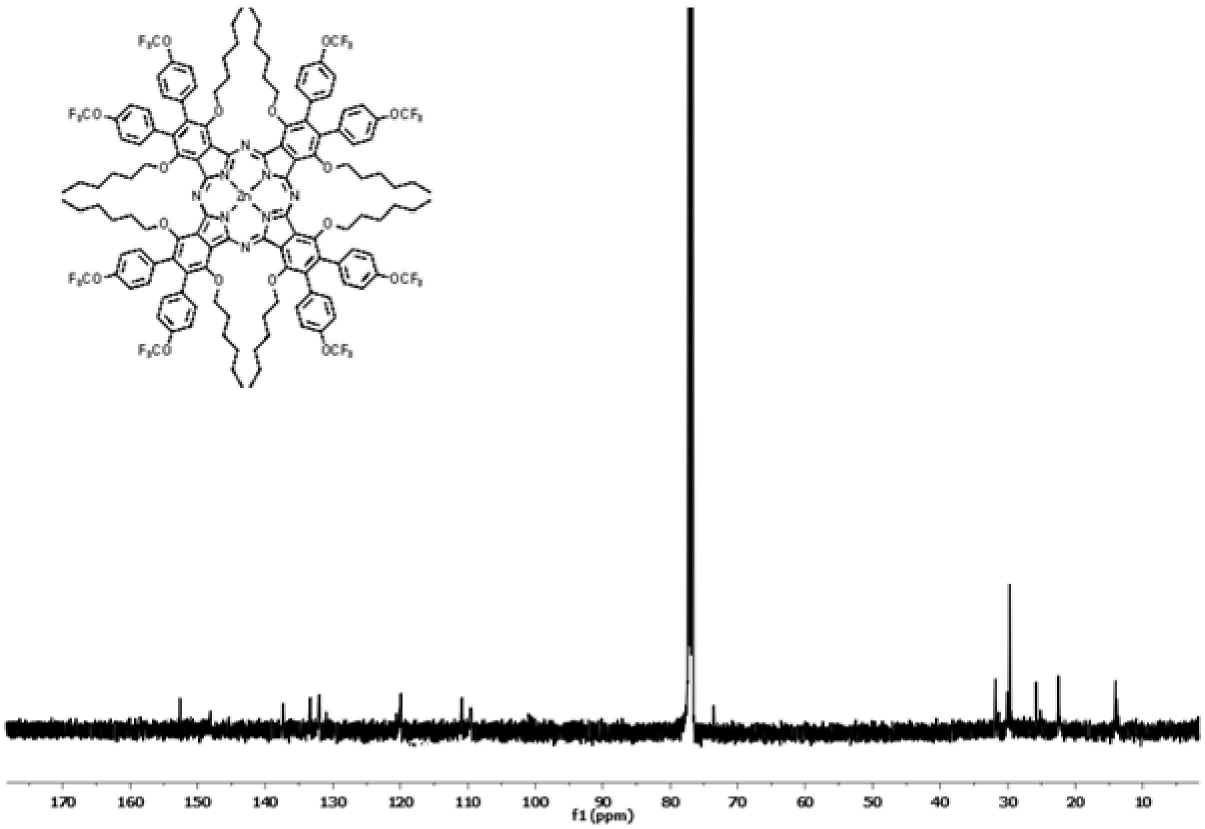

Figure S8. ^{13}C-NMR spectrum of compound **3b** in $CDCl_3$.

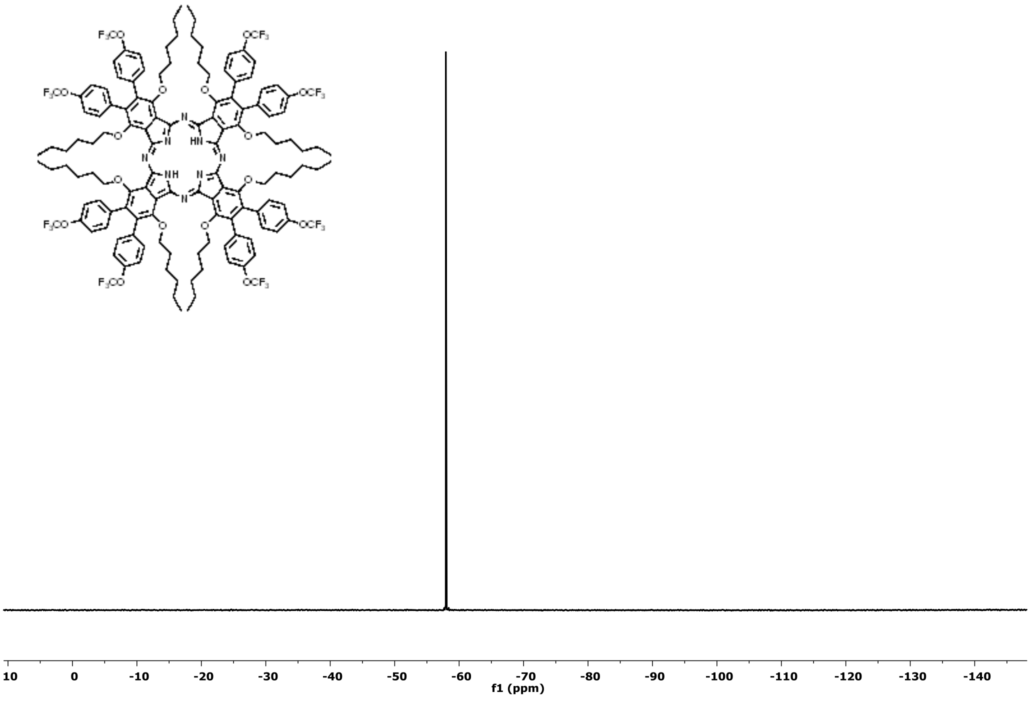

Figure S9. ^{19}F-NMR spectrum of compound **3a** in $CDCl_3$.

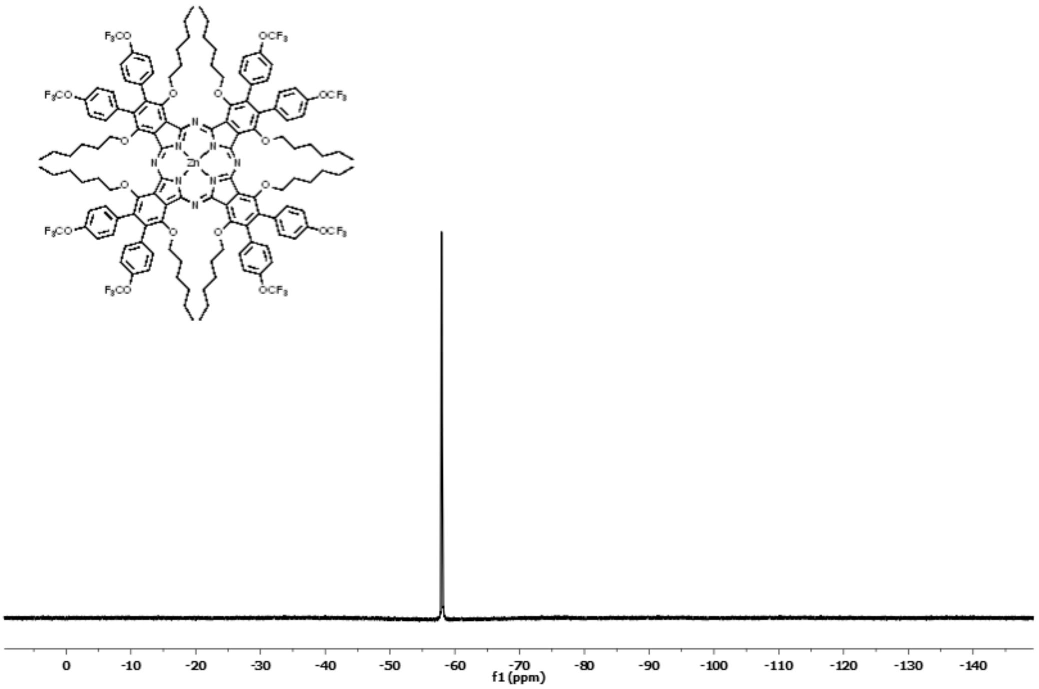

Figure S10. ^{19}F-NMR spectrum of compound 3b in $CDCl_3$.

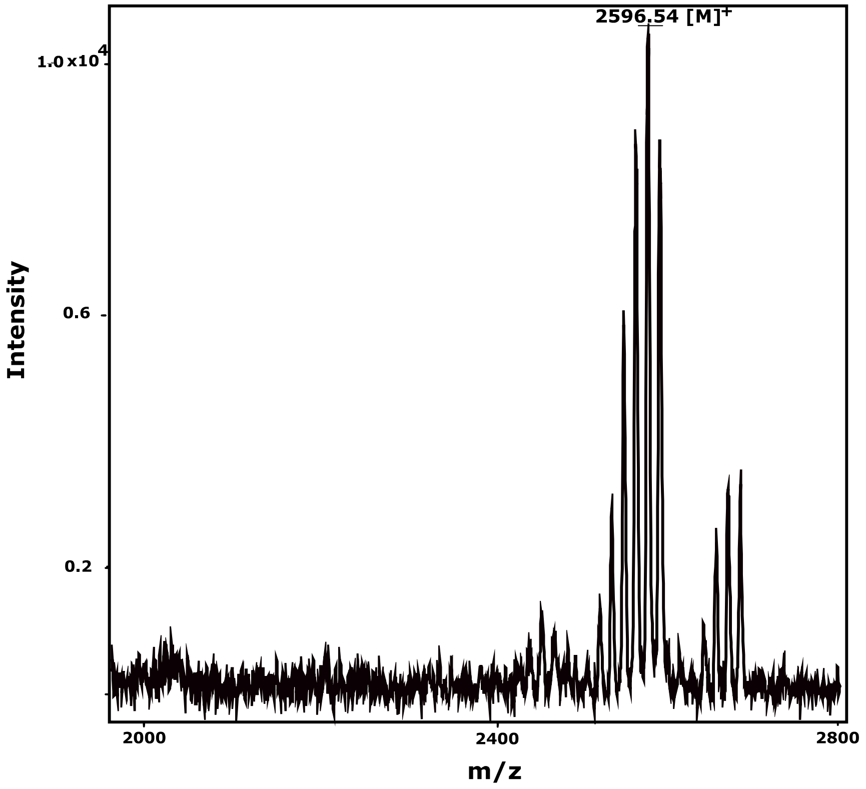

Figure S11. MALDI-TOF spectrum of compound **3a** in $CDCl_3$.

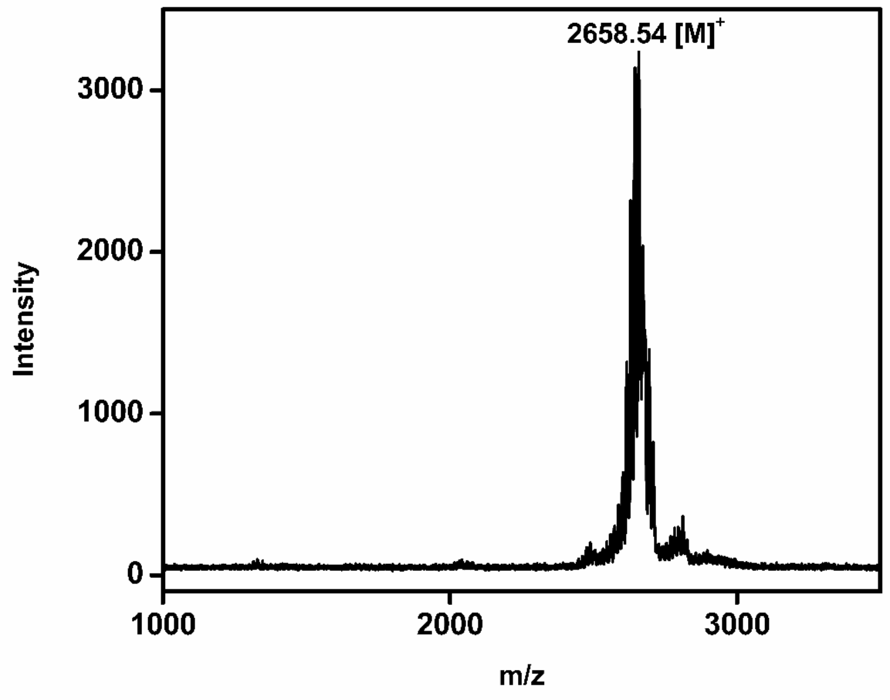

Figure S12. MALDI-TOF spectrum of compound **3b** in $CDCl_3$

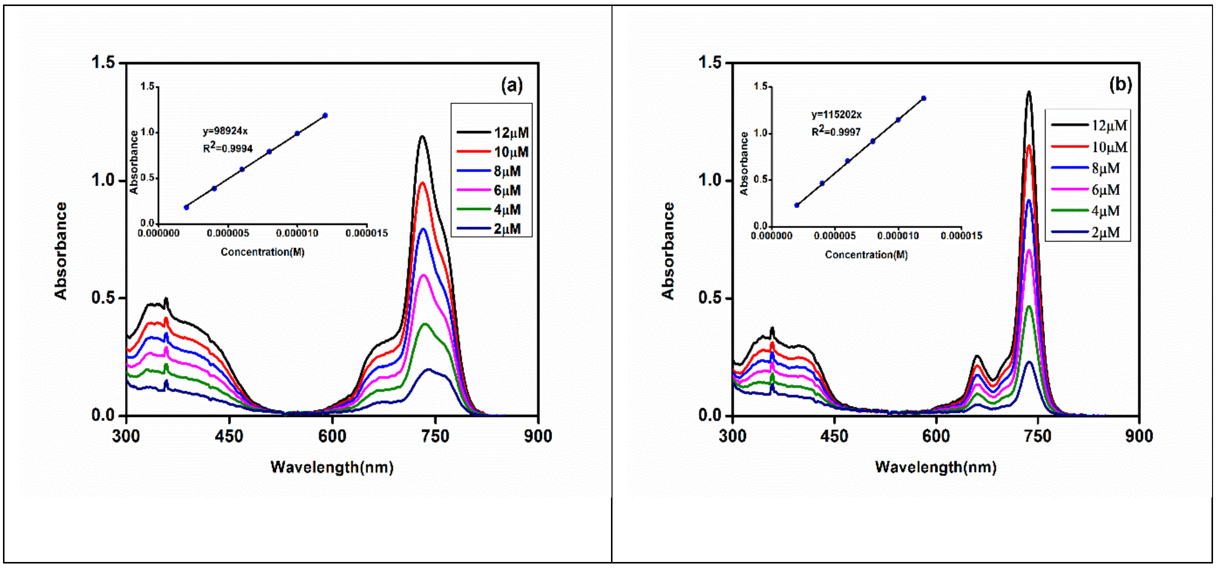

Figure S13. UV–vis spectra of a) **3a** and b) **3b** in DMF at different concentration (C=2-12μM).

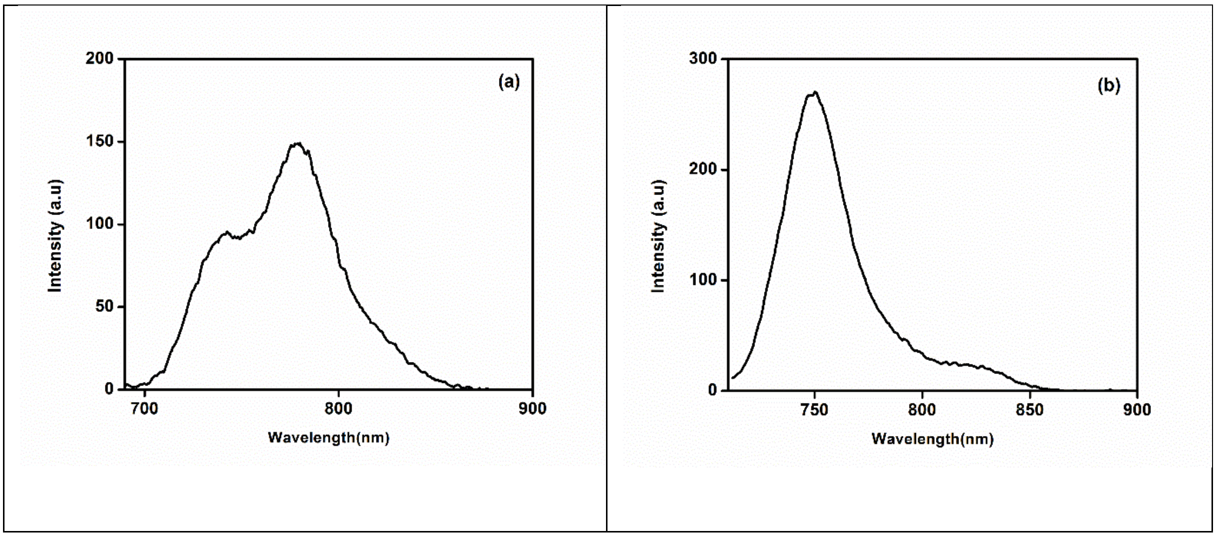

Figure S14. Fluorescence emission spectra of a) phthalocyanine **3a** and b) phthalocyanine **3b** in DMF at $5x10^{-6}$ M. (Excitation wavelength= 686 nm for **3a** and 700 nm for **3b**).

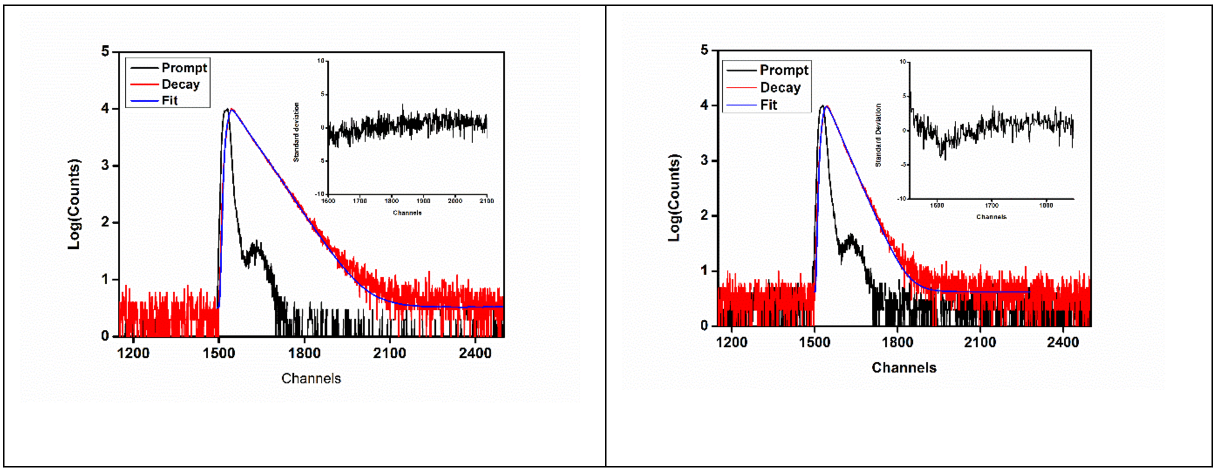

Figure S15. Time correlated single photon counting (TCSPC) trace for a) **3a** (Excitation wavelength=686 nm) and b) **3b** (Excitation wavelength=700 nm) in DMF with residuals.

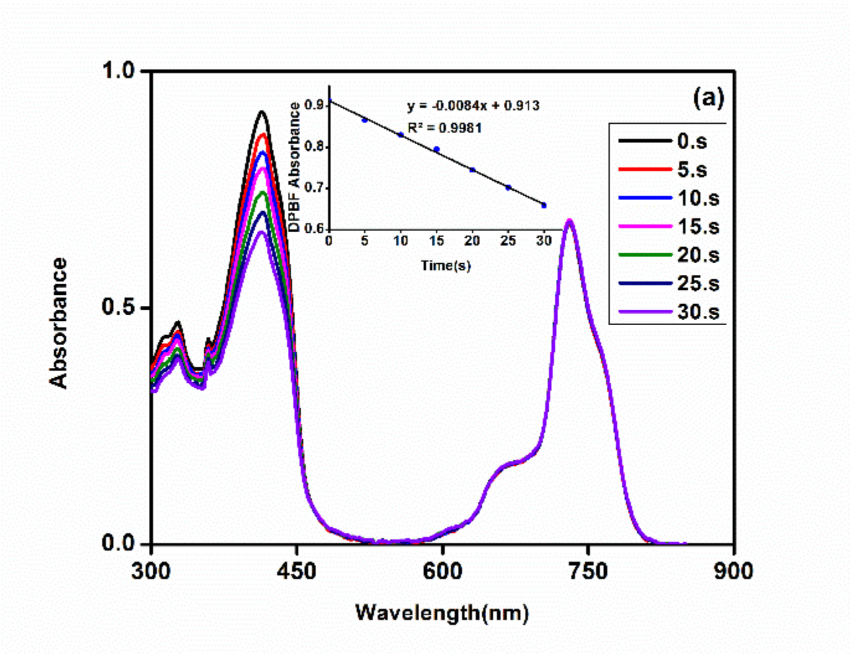

Figure S16. The electronic absorption spectral changes during the determination of singlet oxygen quantum yields. This determination was for **3a** in DMF at a concentration of $1x10^{-5}$ M. (Inset: Plot of DPBF absorbances versus time).

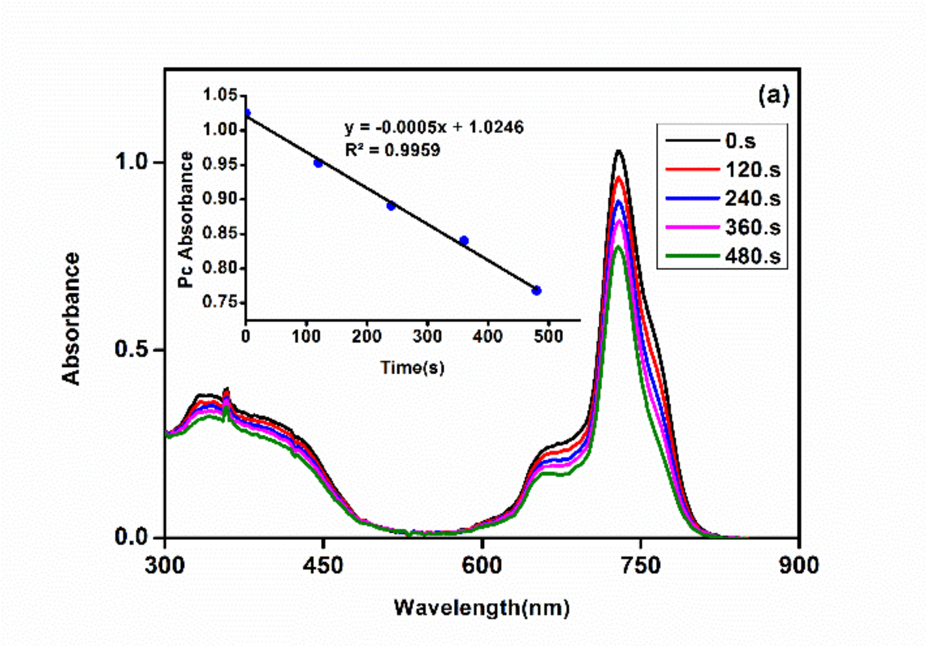

Figure S17. The electronic absorption spectral changes of **3a** in DMF under light irradiation showing the disappearance of the Q-band (Inset: plot of phthalocyanine absorbances versus time).

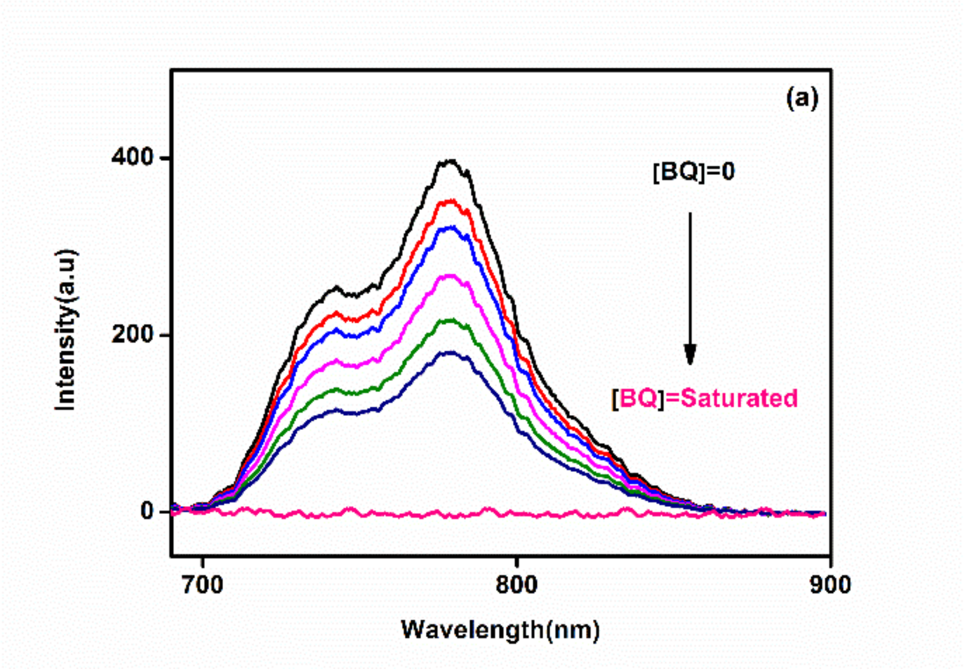

Figure S18. Fluorescence emission spectral changes of **3a** ($1x10^{-5}$M) by the addition of different concentrations of BQ in DMF
